# Building Oral Health Literacy in Adolescence: A Qualitative Exploration of Knowledge and Behaviours in Spain

**DOI:** 10.3390/dj14030176

**Published:** 2026-03-17

**Authors:** Olabarrieta-Zaro Elena, Bernardo-Vilamitjana Natàlia, Figueroa-Marcé Laura, Bastardo-López Zoila, Reig-Garcia Glòria, Pujiula-Blanch Montserrat

**Affiliations:** 1 Primary Health Care Centre Alfons Moré i Paretas, Passeig Marquès de Camps 52–54, 17190 Salt, Spain; 2 Department of Nursing, University of Girona, Emili Grahit 77, 17003 Girona, Spain

**Keywords:** oral health, adolescent, community-based health promotion, health assets

## Abstract

**Background:** Oral health during adolescence is a key determinant of long-term well-being and health equity. Despite widespread recognition of its importance, disparities in knowledge, motivation, and access to care persist. This study was conducted in Salt (Catalonia, Spain), a municipality with a population of approximately 33,000, characterized by a low average household disposable income (€12,512 per capita) and a high proportion of immigrant residents (37.76%). These sociodemographic characteristics may influence adolescents’ oral health behaviour, perceptions, and access to dental care. The study aimed to explore adolescents’ knowledge, habits, and attitudes towards oral health in this context, with barriers and protective factors, to inform community-based health promotion strategies. **Methods:** A qualitative descriptive study was conducted using focus group discussions with Spanish adolescents aged between 12 and 16, following ethical approval and informed consent from legal guardians. Data were systematically analysed using thematic analysis. **Results:** The adolescents had moderate oral health literacy, with basic knowledge of dental caries and prevention, but notable gaps in their knowledge regarding systemic consequences and complementary resources. Oral health behaviours and practices were shaped by social, economic, and normative influences, while parental involvement, community support, and school-based initiatives emerged as key assets for the promotion of oral health. **Conclusions:** While adolescents in Salt show awareness of oral hygiene, structural, motivational, and informational barriers limit comprehensive oral health practices. Interventions should move beyond knowledge-based education towards culturally adapted, participatory, and asset-based approaches to promote sustainable improvements in adolescent oral health.

## 1. Introduction

Oral health constitutes an essential component of overall well-being and is closely linked to the physical, mental, and social health of individuals [1,2,3]. According to the World Health Organization, oral health is defined as a state free from chronic oral and facial pain, oral and throat diseases, infections, ulcers, periodontal diseases, dental caries, tooth loss, and other disorders that affect the oral cavity and general health [4].

Adolescence, generally encompassing the ages between 10 and 19 years, represents a critical stage of human development during which lifelong habits are established, including those related to dental care [5,6]. During this period, adolescents undergo biological, psychological, and social changes that may negatively influence their oral hygiene behaviours, increasing the risk of dental caries and periodontal diseases. The transition from childhood to adulthood may predispose adolescents to neglect oral health, particularly due to a growing desire for independence and the influence of external factors such as family, school, and social environments [6]. Furthermore, factors such as unequal access to dental care services, socioeconomic status, lack of social and family support networks, a diet high in sugars, and the presence of systemic diseases can exacerbate oral health problems within this age group [7,8,9].

Among the main oral health problems affecting adolescents are the high prevalence of dental caries, periodontal disease, dento-maxillary anomalies, molar–incisor hypomineralization and fluorosis, requiring interventions ranging from prophylactic care to restorative treatments [2,10,11]. Although epidemiological studies in Spain show a notable decline in dental caries among adolescents and a high proportion of this population visiting the dentist regularly, the evidence suggests that the adoption of optimal oral hygiene practices is not yet as widespread as it could be. Several studies involving Spanish adolescents indicate that only a subset maintains a toothbrushing frequency considered adequate or attends dental check-ups regularly, and that these behaviours are influenced by lifestyle patterns and environmental factors. This indicates that, while there is a general trend towards engagement in preventive oral health behaviours, gaps persist in the consistent implementation of comprehensive preventive practices and in the overall understanding of oral health, highlighting the relevance of oral health literacy in this population [12,13].

Several studies have shown that, although most adolescents recognize the importance of oral hygiene, significant deficiencies persist in both knowledge and daily practices related to oral health [14,15]. According to Sbricoli [16], while 83% of adolescents brush their teeth at least twice a day, only 7% use dental floss daily. This gap between knowledge and behaviour highlights the need to develop specific interventions. Poor habits could be corrected through health education programmes and the promotion of preventive dental services [17]. In this context, the promotion of oral health among adolescents must be based on a comprehensive approach that considers individual, familial, community, and social factors. Likewise, it is necessary to implement educational and preventive models adapted to the particular characteristics and risks of this developmental stage [18,19]. Designing effective intervention programmes is essential to consolidating healthy habits and improving the quality of life of this population group [20].

In recent years, digital health interventions and social media-based strategies have gained increasing attention as potential health assets for promoting oral health among adolescents. Recent systematic reviews have shown that digital interventions, including mobile applications, text messaging systems, web-based educational platforms, and gamified tools, can significantly improve oral health knowledge, self-efficacy, and preventive behaviours when grounded in behaviour change theories and interactive engagement approaches [21,22,23]. Furthermore, emerging evidence suggests that social media environments, which play a central role in adolescents’ daily communication and identity construction, may function as community-level health assets by reinforcing positive norms, peer modelling, and participatory health promotion dynamics [22,23].

Despite these insights, there remains a clear research gap in Spain regarding the detailed assessment of adolescents’ knowledge, attitudes, and habits related to oral health, particularly in relation to preventive behaviours and oral health literacy. This study addresses this gap by providing new empirical data on how Spanish adolescents understand and manage their oral health. By linking knowledge, attitudes, and actual practices, the study contributes theoretical insight into the relationship between oral health literacy and behaviour in this age group. Furthermore, the findings have practical implications as they can inform the design and implementation of targeted oral health education programmes, guide public health policy, and support Spanish dental health institutions in developing strategies to improve preventive care and overall oral health among adolescents.

## 2. Materials and Methods

### 2.1. Design

A generic qualitative design [24] based on a constructivist–naturalistic approach was employed as it allows the complexity of a phenomenon to be understood from the different viewpoints of the participants [25]. Generic qualitative studies enable researchers to move flexibly across boundaries, use established methodological tools, and develop research designs that align with their epistemological stance, discipline, and specific research questions [26].

### 2.2. Study Setting

The study was conducted in the field of Primary and Community Health Care in a specific city within the Girona Health Region, Catalonia (Spain). The area had a total population of around 33,000 inhabitants, with an average per capita household disposable income of €12,512. Additionally 37.76% of residents were of immigrant origin [27].

### 2.3. Recruitment and Data Collection

The qualitative research consisted of three focus group sessions with adolescents, conducted between May and September 2019. This technique is suitable for exploring participants’ experiences, opinions, desires, and concerns [28]. The sample size was determined using the concept of information power, considering the study objectives, sample specificity, use of established theories, data quality, and analytic strategy [29]. Participants were selected through purposive sampling, prioritizing individuals who were able to provide richer and more relevant information to the study. The study population consisted of adolescents residing in Catalonia, Spain. Catalonia is characterized by considerable cultural and ethnic diversity, and accordingly, the sample included adolescents from both native and migrant backgrounds. All participants, regardless of their ethnic or cultural origin, were residents of the region at the time of the study. Findings should be interpreted within this regional context.

Potential participants were contacted through various social spaces that included adolescents from diverse backgrounds and profiles: a secondary education institute, a youth centre, and a socio-educational project aimed at young people requiring specialized support.

The focus groups followed a semi-structured format based on open-ended questions about oral health. At the beginning of each session, sociodemographic questions were asked to characterize the sample. The questions were carefully developed using input from key stakeholders such as dentists, dental hygienists, and researchers who had previously carried out studies on oral health. Additionally, a thorough literature review was conducted to build a discussion guide designed to explore knowledge, attitudes, and practices related to oral health among adolescents (Table 1).

During the sessions, two researchers shared moderation responsibilities: one served as the moderator, guiding the discussion and ensuring all participants could express their opinions, while the other acted as an observer, taking detailed notes on both verbal and non-verbal communication and summarizing discussions at the end of each session for subsequent analysis.

The focus groups were held in different centres to prioritize participants’ comfort. Each session lasted approximately 40 min and was audio-recorded to ensure accuracy during analysis. All recordings were transcribed verbatim to facilitate data processing.

### 2.4. Data Analysis

Data were analysed by two researchers using content analysis. According to Krippendorff [30], this method is a systematic process for drawing valid and replicable inferences from textual data. The analysis involved several stages:

- Decontextualization, where units of meaning were extracted from their original context.
- Recontextualization, in which these units were situated within an appropriate interpretive framework.
- Categorization, where text fragments were grouped into relevant thematic categories.
- Compilation, through which synthesized information was used to identify patterns and meaningful conclusions.

This process ensured a systematic and rigorous interpretation of the qualitative data, allowing a comprehensive understanding of adolescents’ knowledge, attitudes, and practices concerning oral health [31]. To enhance validity, all emerging themes were discussed and refined by the research team until a consensus was reached [32]. Specifically, the two researchers independently coded the transcripts, and any discrepancies in coding were systematically discussed and resolved through iterative consensus meetings. This process ensured consistent interpretation of the data and enhanced the reliability of the thematic analysis.

To ensure methodological rigour, this study followed the Consolidated Criteria for Reporting Qualitative Research (COREQ) [33], which provides a structured framework to ensure transparency and research quality. These criteria guided the entire process, from study design through reporting. Participant selection procedures emphasized transparency, ensuring that the focus groups were representative of the target population. The roles of the research team members, including those of the moderator and observer, were clearly defined, and the completeness of recordings and transcripts was verified. The content analysis followed a systematic and rigorous framework that ensured objectivity and reliability. Finally, reflexivity was maintained throughout the research process. The researchers responsible for data analysis have prior training and experience in qualitative research methods, including content analysis and thematic coding in health-related studies. In addition, the researchers have academic and professional backgrounds in dentistry and public health, with experience in adolescent oral health promotion. This combined methodological and disciplinary expertise facilitated a nuanced interpretation of the data while remaining attentive to potential preconceptions. To minimize bias, researchers engaged in continuous self-reflection regarding their assumptions about adolescents’ oral health behaviours, and analytic decisions were documented and discussed during iterative consensus meetings. This process helped ensure that interpretations were grounded in participants’ accounts rather than researchers’ expectations.

## 3. Results

A total of 29 adolescents, all aged between 12 and 16 years, participated in the study. Of these, 16 were male and 13 were female. Regarding family origin, participants were of Ghanaian, Senegalese, Mauritanian, Gambian, Malian, Indian, Catalan, and Honduran origin.

**Figure 1:**
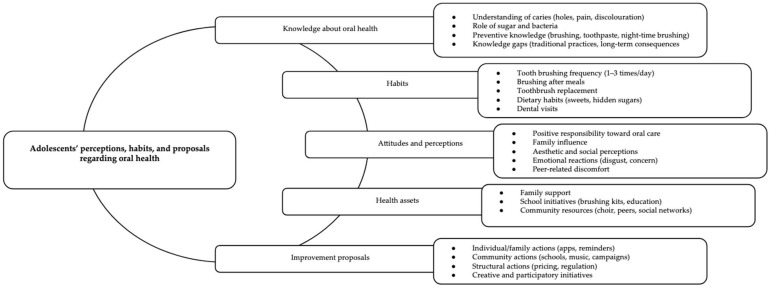
Thematic framework of adolescents’ perceptions, habits, and proposals regarding oral health.

The analysis revealed six general themes related to knowledge, habits, and attitudes toward oral health in the municipality of Salt (Figure 1):

(a) Knowledge about oral health;
(b) Habits;
(c) Attitudes and perceptions;
(d) Health assets;
(e) Improvement proposals.

(a) **Knowledge About Oral Health**

Adolescent understanding of dental caries appears broadly consolidated, especially regarding the visible characteristics and perceived causes. Caries are commonly described as lesions that form holes in teeth, can cause discolouration, and are often associated with pain: “The tooth turns black” (G2); “Cavities are black holes that hurt” (G2).

Many adolescents recognize a connection between sugar consumption and the onset of caries, frequently identifying specific products as potential risk factors: “Eating sugar, especially sweets and chocolate, and also Cola Cao” (G1). Technical explanations also exist, reflecting a general awareness that caries result from bacterial activity affecting dental structures: “The accumulation of bacteria” (G1). Some participants described the process more vividly, personifying bacteria as active agents of decay: “Cavities are tiny little bugs that, when you eat lots of sweets or food and don’t brush your teeth, stay there and start eating away at them” (G5); “The little bugs feed on the sugar on your teeth and make the hole” (G5).

Numerous participants reported having personal experience with caries or knowing individuals affected by them. “When I was 8, I had a cavity and they took out a tooth; after that, I didn’t eat so many sweets” (G2). Similarly, “When I was about 8 or 9, they found a cavity and pulled out my molar, and they told me to be careful. Because it’s bad; it’s not just anything. Since then, I haven’t eaten so many sweets” (G2). The experience of dental caries was occasionally linked to subsequent changes in dietary habits.

Preventive measures are consistently associated with regular tooth brushing, the use of anti-caries toothpaste, and the replacement of toothbrushes every three months. Adolescents demonstrate awareness of brushing duration, utilizing time-management aids such as egg timers or electric toothbrushes with timers. The importance of night-time brushing, especially before bed, is also frequently emphasized. There is further recognition of the need to preserve permanent teeth, acknowledging their irreplaceable nature.

Despite these positive findings, knowledge gaps remain. Traditional oral hygiene practices, such as the use of the Miswak stick, are largely unknown among participants. When asked about alternative or traditional methods of cleaning teeth, several adolescents expressed unfamiliarity or uncertainty: “I’ve never heard of that” (G3); “Is that like a wooden stick?” (G4). Others associated oral hygiene exclusively with commercial dental products, indicating a limited awareness of culturally rooted practices: “You just use a toothbrush and toothpaste… that’s it” (G1).

Some confusion also persists regarding the long-term consequences of caries and the potential systemic implications. While pain and tooth loss are commonly recognized outcomes, broader health connections are less clearly understood. For example, one participant remarked, “If it hurts, they just fix it at the dentist” (G2), suggesting a perception of caries as an isolated and easily reversible condition. Similarly, uncertainty emerged about whether oral diseases could affect overall health: “I don’t think it affects the rest of your body… it’s just the mouth” (G4).

(b) **Habits**

Participants described a range of habits related to oral hygiene, dietary practices, and dental checkups. Tooth brushing habits are generally positive and consistent, with most reporting brushing between one and three times per day, typically in the morning and at night. Some reported brushing more frequently: “I brush my teeth after eating, at least three times, sometimes four” (G2); “I brush twice, sometimes three times” (G2). Brushing after meals was also common, particularly when consuming sticky foods: “If I eat a sticky sweet, I brush my teeth right after” (G2).

Some adolescents mentioned brushing their teeth at school after lunch, facilitated by access to lockers with hygiene materials. The replacement of toothbrushes every three months is a common practice, as noted by one participant: “My brother told me that when brushing your teeth, you should change your toothbrush once every three months” (G3). Brushing the tongue was also mentioned by some.

Regarding dietary habits, most participants understand the connection between consuming sweets and the risk of developing caries. As one participant said, “Sugar, sweets, and not brushing your teeth cause cavities” (G2). Adolescents often demonstrated awareness of the cumulative effect of poor hygiene and sugar intake: “If you drink two glasses of Coca-Cola and stuff yourself with sweets and don’t brush your teeth, well… one day won’t do anything, but if you do it every day, you’ll get cavities” (G2). Despite this awareness, the consumption of these foods occasionally occurs. Some admitted moderate indulgence or routines linked to training schedules: “At home I have a box of sweets, but I only eat them on Tuesdays, because I have training until late, and they help you keep going: they have sugar” (G2), while others described less structured behaviour: “I eat sweets just because I feel like it” (G4). Others reflected on alternative snacks: “We don’t have sweets at home, but I eat junk food… I stop by the bakery and buy myself a croissant” (G2).

Several participants demonstrated an understanding of hidden sugars in everyday products and questioned the accuracy of labelling: “There’s sugar in almost every drink: Fanta… and juices? Even juices, even when it says ‘100% natural,’ it’s faker than…” (G2); “On Fanta it says 0%, that it has no sugar, but you look at the label and… or it says 8% orange juice: so what’s the rest?” (G2).

Dental visits were reported as variable. Some adolescents had only attended school dental checkups, while others described regular annual or semi-annual dental examinations. One participant summarized a common recommendation: “You should go to the dentist twice a year” (G3). For several participants, routine dental care had been established from an early age.

(c) **Attitudes and Perceptions**

The participants show a positive and responsible attitude towards oral health care. Many value the support of their parents, who also brush their teeth and encourage healthy habits. As one participant noted, “My family taught me how to eat properly” (G2), while another added, “My mother taught me too, because her teeth are misaligned and she has to take care of them and use dental floss” (G2).

However, more permissive attitudes appear in some cases: a child observes that “many people don’t care about taking care of their teeth.”

Perceptions related to oral health revolve around aesthetics, hygiene, and social responsibility. Some participants expressed particular attention to others’ dental appearance: “I always look at people’s teeth when they’re talking, because when they speak, I’m interested in their mouth” (G3). The importance of maintaining good hygiene from an early age was also emphasized: “If you didn’t brush your teeth when you were little and you ate a lot of sweets, then when you grow up your mouth will be black…” (G3).

Aesthetic perceptions often carried emotional reactions: “When I see someone with a dirty mouth, it makes me angry… It disgusts me” (G3). The children associate clean teeth with making a good personal impression. However, there is some discomfort or fear of offending friends when talking about cavities, which may hinder the transmission of healthy habits among peers.

(d) **Health Assets**

In the discussions of the focus groups, the concept of assets in oral health emerges. The participants identify various community elements that they consider oral health assets. Among these, school initiatives such as the gift of a brushing kit stand out. Community choirs as a vehicle for campaigning are also considered a health asset.

The importance of family support as an oral health asset is highlighted. Several participants described learning and reinforcement of oral hygiene habits within the family setting: “My parents taught me; we used to brush our teeth together” (G3). Early parental involvement was emphasized as a key influence: “When I was three, my mom would come to me and say, ‘Brush your teeth,’ or she’d tell me to watch YouTube videos: ‘you’ll learn quickly that way’” (G3). Another participant recalled learning through interactive methods at home: “When I learned how to do it, my parents bought me an electric toothbrush and showed me how to use it. It played a little tune, and I had to keep brushing until the music ended” (G3).

Social networks are also mentioned as a potential resource and oral health asset for people in this age group, offering opportunities for learning and positive modelling among peers.

(e) **Proposals for Improvement Actions**

As measures that people can take individually, participants suggested strategies focused on personal and family responsibility. They emphasized the importance of parental involvement and education, for instance, by explaining to parents the need to change toothbrushes regularly and encouraging brushing after eating sweets. Some proposed practical tools such as a brushing guide or reminders through technology. One participant imagined “a mobile app that says ‘Go brush your teeth,’ counts two minutes, and tells you what you can eat” (G5).

As measures that can be taken by the community, participants proposed a wide range of creative and participatory actions. They suggested “making a TV commercial showing how dangerous it can be and what the consequences are” (G2) and using “catchy songs with a rhythm that sticks in your head, saying things about brushing” (G2). Schools were seen as key promoters of oral health, with ideas such as “making groups and going to preschool classes to explain it, because they really listen to us” (G2) or “using a melody from a popular song and changing the lyrics so children brush their teeth while it plays” (G2).

Other proposals focused on visibility and awareness in everyday spaces: “put photos or magnets on the fridge,” or “put positive messages on the fridge with the steps for brushing your teeth” (G2). Environmental and marketing interventions were also discussed, including “putting messages on sweet wrappers like ‘This has sugar; be careful with your teeth’” (G2) and “giving out toothbrushes: it works, because many little kids now brush their teeth at school” (G2).

Structurally, participants supported measures aligned with health promotion principles, such as increasing the price of sweets and eliminating bulk sales. They also envisioned collective creative projects such as “making a story together about a child who didn’t brush his or her teeth and what happened afterward” (G5) or community engagement initiatives like “setting up a stand at the Friday market to sell the magnets and the story” (G5).

## 4. Discussion

This study explored adolescents’ knowledge, habits, and attitudes towards oral health, revealing important insights with practical implications for public health interventions. Our findings are consistent with previous studies conducted in European and Mediterranean adolescent populations, which report adequate awareness of oral hygiene importance, but suboptimal adherence to preventive practices [16,34].

With regard to knowledge, most adolescents demonstrate a basic understanding of what dental caries are, how they develop, and how they can be prevented, reflecting a moderate level of oral health literacy. Similar levels of basic oral health knowledge have been reported in previous qualitative and quantitative studies among adolescents [16]. Nevertheless, important gaps persist, including limited awareness of traditional practices such as the *Miswak* stick and only a partial understanding of the systemic consequences of oral diseases. While limited knowledge of the systemic impact of oral diseases is widely documented in adolescent populations, the lack of familiarity with traditional practices contrasts with findings from studies conducted in cultural contexts where such practices are more commonly integrated into health education [16]. Moreover, not all cultural traditions of health education place equal emphasis on oral health [16], which may contribute to these disparities. These findings suggest that educational programmes should move beyond the transmission of basic information and instead provide content that is developmentally appropriate and culturally responsive. In line with previous intervention studies, health promotion interventions grounded in behaviour change theories and incorporating interactive methods and community participation have been shown to be more effective in enhancing both knowledge and clinical outcomes [19].

Dental hygiene habits among adolescents are generally satisfactory with regard to tooth brushing, reflecting regular practice and a positive attitude towards oral cleanliness. This finding aligns with earlier studies reporting high rates of tooth brushing among adolescents [16,32]. However, the use of dental floss and the regularity of dental check-ups remain highly inconsistent, particularly among younger groups and those with limited access to healthcare services. This divergence between simple and more complex preventive behaviours that has been consistently described in the literature highlights a persistent gap between knowledge and actual practice [16], underscoring the need for targeted educational interventions that promote comprehensive and sustained oral care routines.

The findings indicate that adolescents’ oral health behaviours are shaped by a combination of individual, familial, and social factors. Positive attitudes towards oral hygiene, reinforced by parental support and social networks, function as key protective elements. At the same time, permissive norms and the perception that peers place limited importance on dental care reveal persistent barriers to maintaining consistent healthy behaviours. While previous studies have emphasized the role of socioeconomic status, diet, and family environment in shaping adolescent oral health behaviours [9], the present findings further highlight the influence of peer norms, self-image, and growing autonomy during adolescence. This tension between knowledge and practice reflects the broader influence of social determinants and underscores the impact of peer dynamics and growing autonomy during adolescence on oral health outcomes. Moreover, adolescents display a strong sense of self-image and social responsibility linked to dental aesthetics. This finding goes further than prior research by suggesting that aesthetic and social motivations may represent an underutilized entry point for promoting oral health behaviours in this age group.

One of the most relevant contributions of this study is the identification of health assets, a concept that is still relatively unexplored in the field of oral health. Unlike most previous studies, which have primarily adopted a deficit-based approach focusing on risk factors and negative outcomes, adolescents in this study identified school settings, community initiatives, social networks, and family support as valuable resources for promoting oral health. This perspective aligns with the health assets model, which emphasizes the resources available within communities to enhance individual and collective health and well-being [35]. By applying this framework to adolescent oral health, the present study extends existing literature and supports a shift toward more sustainable, strengths-based health promotion strategies. Additionally, the identification of social media as a key resource for this age group underscores the importance of incorporating digital platforms into health promotion programmes. In recent years, digital health interventions and social media-based strategies have gained increasing attention as potential health assets for promoting oral health among adolescents. Recent systematic reviews have shown that digital interventions, including mobile applications, text messaging systems, web-based educational platforms, and gamified tools, can significantly improve oral health knowledge, self-efficacy, and preventive behaviours, particularly when grounded in behaviour change theories and interactive engagement approaches [21,22,23]. Furthermore, given the central role of social media both in adolescents’ daily communication and in the construction of their identities, these environments may function as community-level health assets by reinforcing positive norms, peer modelling, and participatory health promotion dynamics [22,23]. These findings support the integration of evidence-based digital strategies within comprehensive, strengths-based oral health promotion frameworks.

Proposals for improvement actions should thus focus on multi-level interventions addressing individual behaviour change and community engagement. Efforts to educate parents and caregivers about critical practices, such as regular toothbrush replacement and brushing after eating sweets, combined with school and media campaigns, could improve oral hygiene adherence. These recommendations are consistent with previous public health strategies emphasizing integrated, multi-sectoral approaches to oral health promotion [19,36]. Structural strategies, such as regulating the accessibility and marketing of sugary products, would complement these behavioural interventions by addressing underlying determinants of oral health.

Limitations of the study include the relatively small sample size and the regional focus on a specific health area, which might limit the generalizability of the findings. In addition, the focus group design does not allow for the examination of changes in knowledge, attitudes, and behaviours over time. The interactive nature of focus groups may have influenced participants’ responses due to group dynamics, social desirability, or peer pressure. Although the study was conducted in Catalonia (Spain), the inclusion of adolescents from diverse cultural and migrant backgrounds reflects the demographic characteristics of this region; however, the results should be interpreted within this particular regional and sociocultural context. Furthermore, the data were collected between May and September 2019, which may limit the timeliness of the findings, as social and health-related contexts may have evolved since then. Future research should explore more contemporary contexts in Spain, as updated data could provide new insights and additional empirical evidence. It would also be valuable to explore larger, more diverse populations and to assess the long-term impact of proposed interventions. Experimental studies evaluating the effectiveness of digital health tools and culturally tailored programmes could further strengthen the evidence base for comprehensive adolescent oral health promotion.

In conclusion, this qualitative investigation provides a nuanced understanding of adolescent oral health perspectives and both confirms and extends previous research by reinforcing well-documented gaps between knowledge and practice while offering new qualitative insights into the social and community assets that may support effective, context-sensitive oral health promotion strategies.

## 5. Conclusions

Adolescents are found to have basic knowledge of oral health and consistent tooth brushing habits, yet gaps remain regarding flossing, dental check-ups, systemic consequences of oral diseases, and traditional hygiene practices. Oral health behaviours are influenced by self-image, peer norms, and social and economic factors, while parental support, school initiatives, and community networks act as protective factors.

Participants identified multiple health assets and proposed strategies at individual, family, and community levels, including educational programmes, digital reminders, media campaigns, and structural measures targeting sugary foods. These findings highlight the limitations of knowledge-only interventions and underscore the potential of culturally adapted, participatory, and asset-based approaches.

Integrating individual, familial, and community resources in oral health promotion can improve literacy, foster consistent preventive practices, and support long-term well-being. Future research should explore larger, diverse populations and evaluate the effectiveness of multi-level, context-sensitive interventions.

Importantly, existing community resources, such as school-based programmes, family engagement structures, local health services, and digital communication platforms, should be systematically integrated into municipal and regional oral health policies to ensure sustainable, equity-oriented prevention strategies.

## Figures and Tables

**Table 1:** Focus Group Guide.

Do you know what cavities are?
Do you know what causes them?
Do you know how they can be prevented?
What do you do to maintain good oral and dental health?
How important is oral care to you?
What importance would you give to your children’s oral care?
How can we identify resources and potential interventions?
What can be done?
What do you think you could contribute?

## Data Availability

The data that support the findings of this study are available from the corresponding author, Glòria Reig, upon reasonable request (gloria.reig@udg.edu). Access to the data may be subject to ethical approval and data sharing agreements to protect participant confidentiality.
